# Supplementary material for: Impact of inherent energy barrier on spin-orbit torques in magnetic-metal/semimetal heterojunctions
Source: Nat Commun. 2023 Aug 25;14:5187. doi: 10.1038/s41467-023-40876-9 (PMC10457350; doi:10.1038/s41467-023-40876-9)
Supplement: Supplementary file 1 — Supplementary Information [file 41467_2023_40876_MOESM1_ESM.pdf]

Supplementary Information

Impact of inherent energy barrier on spin-orbit torques in magnetic-metal/semimetal heterojunctions

Tenghua Gao[a],[1,2,3] Alireza Qaiumzadeh[a],[4] Roberto E. Troncoso[a],[4,5] Satoshi Haku,[2] Hongyu An,[6] Hiroki Nakayama,[2] Yuya Tazaki,[2] Song Zhang,[3] Rong Tu,[3] Akio Asami,[2] Arne Brataas,[4] and Kazuya Ando[b2,1,7]

[1]*Keio Institute of Pure and Applied Science, Keio University, Yokohama 223-8522, Japan*

[2]*Department of Applied Physics and Physico-Informatics, Keio University, Yokohama 223-8522, Japan*

[3]*State Key Laboratory of Advanced Technology for Materials Synthesis and Processing, Wuhan University of Technology, Wuhan, China*

[4]*Center for Quantum Spintronics, Department of Physics, Norwegian University of Science and Technology, NO-7491 Trondheim, Norway*

[5]*School of Engineering and Sciences, Universidad Adolfo Ibáñez, Santiago, Chile*

[6]*College of New Materials and New Energies, Shenzhen Technology University, Shenzhen 518118, China*

[7]*Center for Spintronics Research Network, Keio University, Yokohama 223-8522, Japan*

[a] These authors contributed equally

[b] Correspondence and requests for materials should be addressed to ando@appi.keio.ac.jp

Supplementary Note 1: Structural properties of $Bi_{0.1}Sb_{0.9}$ films

Although advances in spin-orbitronics have developed the framework to understand spin-orbit torques, the impact of inherent energy barriers at metallic interfaces on the spin-orbit torques has been overlooked. At an interface with weak spin-orbit coupling, the spin memory loss is negligible, and spin transport is mainly determined by the spin mixing conductance. This is the case for our $Ni_{81}Fe_{19}/Bi_{0.1}Sb_{0.9}$ heterojunctions. When the spin transport is governed by the spin mixing conductance, two physical processes are present: 1. The incident spins transmit across the interface and absorbed by the magnetic moment; 2. The incident spins briefly precess around the magnetization when reflecting off the interface. The first process is characterized by the real part of the spin mixing conductance associated with the damping-like (DL) torque generation, while the second process is characterized by the imaginary part of the spin mixing conductance associated with the field-like (FL) torque generation. Up to now, it remains elusive how an ultrathin carrier depletion layer influences these two parameters, and consequently on the DL and FL torque generation.

Since our study focuses on the above issue, the effective bulk spin Hall angle of around 0.5 in $Bi_{0.1}Sb_{0.9}$ is just a minor point of this work. The choice of the semimetal $Bi_{0.1}Sb_{0.9}$ with the high conductivity and large bulk spin current generation efficiency is a prerequisite for this study; the composition is not optimized to achieve a large spin Hall angle but optimized to explore the impact of the inherent energy barrier on the bulk dominated spin-orbit torques. In general, materials with low conductivities can show large spin Hall angles, such as β-W. We note that the electrical properties of $Bi_{1-x}Sb_x$ binary alloys are highly dependent on the composition. When $0.07 < x < 0.22$, $Bi_{1-x}Sb_x$ is a highly resistive narrow-gap semiconductor. Out of this composition range, $Bi_{1-x}Sb_x$ alloys are semimetals and become more and more conductive as the composition is close to pure Sb. When $0.07 < x$, the nontrivial surface states are expected to be present [1]. Besides, the spin-orbit coupling is expected to be weaker for Sb rich $Bi_{1-x}Sb_x$ alloys [2]. By scarifying the strength of spin-orbit coupling, but maintaining a high bulk conductivity, we use semimetal $Bi_{0.1}Sb_{0.9}$ as a spin source material in the present work. We also note that a high atomic percentage of Bi in BiSb or BiSe binary alloys often leads to a rough interface when depositing $Ni_{81}Fe_{19}$/BiSb or $Ni_{81}Fe_{19}$/BiSe bilayers, causing strong interdiffusion. The Sb rich BiSb alloy with a Bi to Sb atomic ratio of 1:9 allows us to achieve a much better $Ni_{81}Fe_{19}$/BiSb interface.

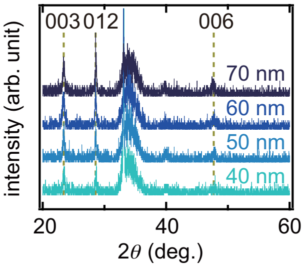

Supplementary Figure 1. X-ray diffraction profiles of the sputtered $Bi_{0.1}Sb_{0.9}$ films with the thickness $t = 40$, 50, 60, and 70 nm. The unlabeled peaks are from the thermally oxidized Si substrate.

To investigate the structural properties of the $Bi_{0.1}Sb_{0.9}$ films with different thickness t, we performed x-ray diffraction measurements. As shown in Supplementary Fig. 1, the $Bi_{0.1}Sb_{0.9}$ films fabricated by sputtering generally have a polycrystalline feature. The nearly same (003)/(012) peak height ratio indicates that the preferred crystalline orientations in the $Bi_{0.1}Sb_{0.9}$ film remain unchanged upon varying the film thickness. This result confirms that the structural properties of the $Bi_{0.1}Sb_{0.9}$ films are independent of the thickness, consistent with the thickness-independent transport properties (see Supplementary Note 3).

In Supplementary Fig. 2(a), we show a magnified TEM image of the interface region for the $Ni_{81}Fe_{19}/Bi_{0.1}Sb_{0.9}$ bilayer. In the magnified image, the interface is ambiguous due to the nearly indistinguishable contrast between $Ni_{81}Fe_{19}$ and $Bi_{0.1}Sb_{0.9}$, as well as the polycrystalline nature of the material. However, this result also shows that two regions with different crystallographic orientations are distinguishable, suggesting the formation of a well-defined $Ni_{81}Fe_{19}/Bi_{0.1}Sb_{0.9}$ interface. We have also employed the high-angle annular dark-field scanning transmission electron microscopy (HAADF-STEM) technique to enhance the contrast between $Ni_{81}Fe_{19}$ and $Bi_{0.1}Sb_{0.9}$, as shown in Supplementary Fig. 2(b). This result suggests a clear $Ni_{81}Fe_{19}/Bi_{0.1}Sb_{0.9}$ interface, even though the thickness of the $Bi_{0.1}Sb_{0.9}$ layer is as much as 50 nm. Here, the effective demagnetization field $M_{\rm eff}$ of the $Ni_{81}Fe_{19}/Bi_{0.1}Sb_{0.9}$ bilayer is found to be 0.834 T (see also Supplementary Note 7), which is slightly larger than 0.805 T of $Ni_{81}Fe_{19}$/Pt bilayers [3], supporting that the intermixing is not significant at the $Ni_{81}Fe_{19}/Bi_{0.1}Sb_{0.9}$ interface.

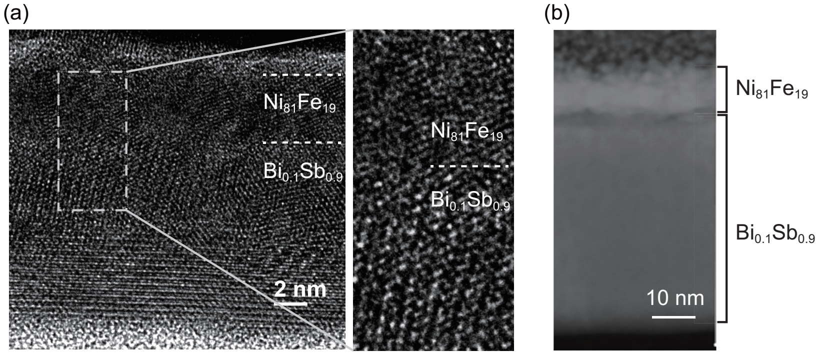

Supplementary Figure 2. (a) A high resolution cross-sectional TEM image (left panel) of the $Ni_{81}Fe_{19}$ (6 nm)/$Bi_{0.1}Sb_{0.9}$ (10 nm) bilayer. The right panel shows an enlarged image of the interface region indicated by the white dashed rectangle in the $Ni_{81}Fe_{19}$/$Bi_{0.1}Sb_{0.9}$ bilayer. (b) A cross-sectional HAADF-STEM image of the $Ni_{81}Fe_{19}$ (6 nm)/$Bi_{0.1}Sb_{0.9}$ (50 nm) bilayer.

Supplementary Note 2: Analysis of spin-torque ferromagnetic resonance signals

Magnetic field H dependence of a spin-torque ferromagnetic resonance (ST-FMR) spectrum can be decomposed into symmetric ($L_{\mathrm{sym}}(H) = W^2/[(\mu_0 H - \mu_0 H_{\mathrm{FMR}})^2 + W^2]$) and antisymmetric ($L_{\mathrm{asym}}(H) = W(\mu_0 H - \mu_0 H_{\mathrm{FMR}})/[(\mu_0 H - \mu_0 H_{\mathrm{FMR}})^2 + W^2]$) functions [3, 4]: $V_{\mathrm{mix}} = V_{\mathrm{s}} L_{\mathrm{sym}}(H) + V_{\mathrm{a}} L_{\mathrm{asym}}(H)$. We measured in-plane magnetic field angle θ dependence of the ST-FMR for a representative $Ni_{81}Fe_{19}$(6 nm)/$Bi_{0.1}Sb_{0.9}$(30 nm) device to check a possible lateral non-uniformity of the RF current. Here, the ST-FMR device is a rectangular strip with a width of 7 μm and a length of 49 μm, as described in Methods section. Supplementary Figure 3 shows the θ dependence of the symmetric V_{s} and antisymmetric V_{a} components of the ST-FMR signal measured at the RF frequency f of 7 GHz. This result shows that both V_{s} and V_{a} follow the $\cos\theta \sin 2\theta$ angular dependence, which is consistent with the ST-FMR induced by an out-of-plane effective field due to a DL torque and an in-plane effective field due to the sum of a FL torque and an Oersted field [4]. In particular, the vanishingly small $\sin 2\theta$ component in V_{s} suggests that an out-of-plane Oersted field due to a non-uniform current is negligible in our ST-FMR device.

Non-uniform current distribution within the $Ni_{81}Fe_{19}$ layer can give rise to an in-plane Oersted field, in addition to an in-plane Oersted field created by the current flow in the $Bi_{0.1}Sb_{0.9}$ layer. In our previous study, we successfully simulated the non-uniform current

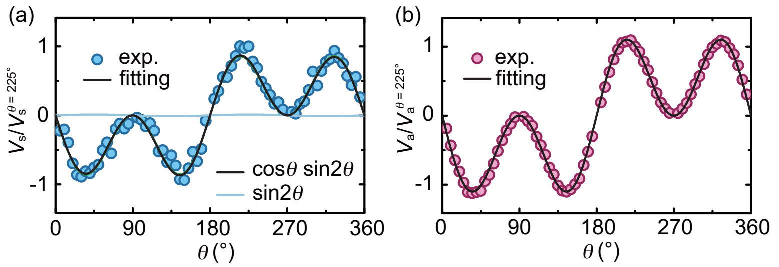

Supplementary Figure 3. In-plane magnetic field angle θ dependence of the (a) symmetric V_{s} and (b) antisymmetric V_{a} components of the ST-FMR signal for the $Ni_{81}Fe_{19}$(6 nm)/$Bi_{0.1}Sb_{0.9}$(30 nm) device. $V_{\mathrm{s(a)}}^{\theta=225^\circ}$ is the symmetric(antisymmetric) component the ST-FMR signal measured at $\theta = 225^\circ$. The solid circles are the experimental data, and the solid curves are the fitting results using functions proportional to $\cos\theta \sin 2\theta$.

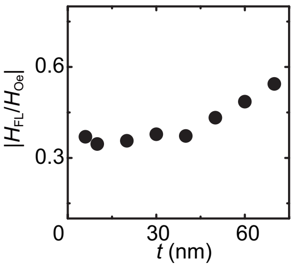

Supplementary Figure 4. The ratio $|H_{\rm FL}/H_{\rm Oe}|$ as a function of the $Bi_{0.1}Sb_{0.9}$-layer thickness t for the $Ni_{81}Fe_{19}$(6 nm)/$Bi_{0.1}Sb_{0.9}$(t) bilayer.

distribution in a $Ni_{81}Fe_{19}$ layer using parameters determined from real samples [5]. The calculation demonstrates that the in-plane Oersted field, resulting from the inhomogeneous current distribution, is more pronounced in thick $Ni_{81}Fe_{19}$ films. The result shows that the in-plane Oersted field per unit electric field due to the non-uniformity of the RF current in a $Ni_{81}Fe_{19}$(7.5 nm) film is estimated to be $\mu_0 H_{\rm Oe}/E = 1.4\times10^{-4}$ μTV^{-1}m. This value is an order of magnitude smaller than $\mu_0 H_{\rm Oe}/E = 14.5\times10^{-4}$ μTV^{-1}m in a $Ni_{81}Fe_{19}$(6 nm)/$Bi_{0.1}Sb_{0.9}$(10 nm) bilayer, where the in-plane Oersted field is assumed to be generated by the current flowing in the $Bi_{0.1}Sb_{0.9}$ layer. As the in-plane Oersted field due to the current flow in the $Bi_{0.1}Sb_{0.9}$ layer increases with the thickness of the $Bi_{0.1}Sb_{0.9}$ layer, the Oersted field due to the non-uniform current in the $Ni_{81}Fe_{19}$ layer can be considered to be negligible in the devices used in this work. Therefore, it is unlikely to significantly affect the accuracy of the FL-SOT efficiency determined in this study. In Supplementary Fig. 4, we show the ratio of $H_{\rm FL}$ to $H_{\rm Oe}$ as a function of the $Bi_{0.1}Sb_{0.9}$ thickness t, where $H_{\rm Oe}$ is assumed to be generated by the RF current in the $Bi_{0.1}Sb_{0.9}$ layer. This result shows that the magnitudes of $H_{\rm FL}$ and $H_{\rm Oe}$ are of the same order in the $Ni_{81}Fe_{19}$/$Bi_{0.1}Sb_{0.9}$ bilayers.

At $\theta = 45^\circ$, the symmetric and antisymmetric components are expressed as

$$V_{\rm s} = \frac{I_{\rm RF}\Delta R}{2}\mu_0 H_{\rm DL}\frac{\gamma(H_{\rm FMR}+M_{\rm eff})\mu_0 H_{\rm FMR}}{2\sqrt{2}\pi f W(2H_{\rm FMR}+M_{\rm eff})}, \tag{S1}$$

$$V_{\rm a} = \frac{I_{\rm RF}\Delta R}{2}(\mu_0 H_{\rm FL}+\mu_0 H_{\rm Oe})\frac{(H_{\rm FMR}+M_{\rm eff})}{\sqrt{2}W(2H_{\rm FMR}+M_{\rm eff})}. \tag{S2}$$

Here, γ is the gyromagnetic ratio. The FMR field $H_{\rm FMR}$ and linewidth W are obtained by fitting measured ST-FMR spectra using $V_{\rm mix} = V_{\rm s}L_{\rm sym}(H) + V_{\rm a}L_{\rm asym}(H)$, while the demagnetization field $M_{\rm eff}$ is extracted from the fitting of f as a function of $H_{\rm FMR}$ using Kittel formula. The quantitative characterization of DL and FL effective fields, $H_{\rm DL}$ and

$H_{\rm FL}$, using equations (S1) and (S2) requires to quantify the values of the radio frequency current $I_{\rm RF}$ flowing in devices and the resistance change ΔR of the $\rm Ni_{81}Fe_{19}/Bi_{0.1}Sb_{0.9}$ bilayer film due to the anisotropic magnetoresistance (AMR).

We measured the transmission S_{21} and reflection S_{11} coefficients of the ST-FMR device using a Vector Network Analyzer (VNA), which allows us to determine $I_{\rm RF}$ flowing in the $\rm Ni_{81}Fe_{19}/Bi_{0.1}Sb_{0.9}$ bilayer devices [6]. To estimate the insertion loss, IL, through our RF circuit, we connected all the components including two nominally congruent bias tees and two GSG probes using cables with characteristic impedance of 50 Ω. Meanwhile, the two GSG probes were connected by a "through" with a similar shape as the ST-FMR electrode. Assuming that each half of the circuit at either side of the "through" is the same, we obtain the insertion loss of our ST-FMR circuit from the measured S_{21}: $IL = -10\log_{10}|S_{21}|$ (dB). For the characterization of the reflection coefficient of each ST-FMR device, we first calibrate the system composed of the GSG probe and cable using standard calibration kits. Then, the GSG probe was made contact with the ST-FMR device, and the return loss, RL, was measured using VNA, which is calculated as $RL = 20\log_{10}|S_{11}|$ (dB). By applying the charge conservation law, the current passing through the device is expressed as $I_{\rm RF} = I_{\rm In} - I_{\rm Ref} = I_{\rm In}(1-|S_{11}|)$, where $I_{\rm In}$ is the incident current to the device, and $I_{\rm Ref}$ is the reflected current from the device. Therefore, the RF current flowing in the device as a function of the applied RF power is expressed as

$$I_{\rm RF} = (1-|S_{11}|)\sqrt{\frac{2\times 10^{[P({\rm dBm})+IL({\rm dB})-30]/10}}{Z_0}}. \tag{S3}$$

Here, P is power provided by the RF generator during the ST-FMR measurements, and Z_0 is the impedance of the co-planar waveguide used as the electrode of the ST-FMR devices. To verify the reliability of this calibration method, we characterized the power-dependence of the RF current for a standard $\rm Pt/Ni_{81}Fe_{19}$ sample using two different approaches: the method described above and the Joule heating method [5]. We found that the RF current for each power obtained by these two approaches is quite consistent, with an error less than 10%.

For the measurements of the resistance change due to the AMR, the $\rm Ni_{81}Fe_{19}/Bi_{0.1}Sb_{0.9}$ bilayer films, patterned into Hall bars, with various $\rm Bi_{0.1}Sb_{0.9}$ thicknesses t were fabricated in the same batch as the corresponding rectangular strips used for the ST-FMR measurements. In Supplementary Fig. 5(a), we show a typical AMR curve for the $\rm Ni_{81}Fe_{19}$(6

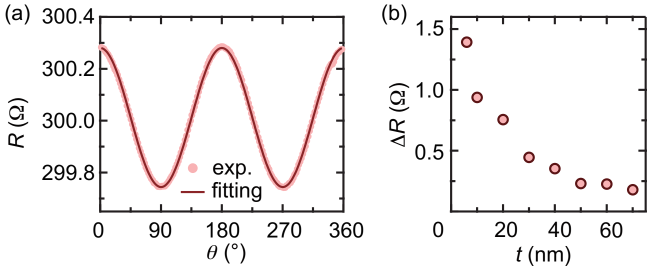

Supplementary Figure 5. (a) A typical in-plane AMR measurement on the $Ni_{81}Fe_{19}$(6 nm)/$Bi_{0.1}Sb_{0.9}$(10 nm) bilayer film under an external magnetic field H of 2000 Oe. The horizontal axis θ refers to the angle between the direction of H and the applied current. (b) The obtained AMR amplitude ΔR of the ST-FMR devices as a function of the $Bi_{0.1}Sb_{0.9}$ thickness t.

nm)/$Bi_{0.1}Sb_{0.9}$(10 nm) bilayer film under an in-plane external magnetic field of 2000 Oe. The same measurements have been performed for all the samples with t ranging from 6 to 70 nm. Here, the resistance change of the bilayer, ΔR, due to the AMR is expressed as [7]

$$\Delta R = \frac{\Delta R_{\mathrm{Ni_{81}Fe_{19}}}}{R_{\mathrm{Ni_{81}Fe_{19}}}} \frac{R}{R_{\mathrm{Ni_{81}Fe_{19}}}} R, \tag{S4}$$

where $\Delta R_{\mathrm{Ni_{81}Fe_{19}}}$ is the resistance change of the $Ni_{81}Fe_{19}$ layer due to the AMR and $R_{\mathrm{Ni_{81}Fe_{19}}}$ is the resistance of the $Ni_{81}Fe_{19}$ layer in the bilayer. R is total resistance of the bilayer. Since $\Delta R_{\mathrm{Ni_{81}Fe_{19}}}/R_{\mathrm{Ni_{81}Fe_{19}}}$ and $R/R_{\mathrm{Ni_{81}Fe_{19}}}$ of the ST-FMR device should be the same as those of the Hall bar when t is the same between the two $Ni_{81}Fe_{19}$(6 nm)/$Bi_{0.1}Sb_{0.9}$(t) structures. Thus, we obtain ΔR of the ST-FMR device with a thickness of t from the measured value of ΔR for the Hall bar with t using the bilayer resistance R of the Hall bar and ST-FMR device. The obtained values of ΔR for the ST-FMR devices is shown in Supplementary Fig. 5(b), which decreases monotonically with increasing the $Bi_{0.1}Sb_{0.9}$ thickness t.

The obtained H_{DL} and H_{FL} with different $Bi_{0.1}Sb_{0.9}$ thicknesses are converted to the DL and FL SOT efficiencies per unit electric field E, defined as $\xi^{E}_{\mathrm{DL(FL)}} = (2e/\hbar)\mu_0 M_{\mathrm{s}} t_{\mathrm{F}} H_{\mathrm{DL(FL)}}/E$, where e is the electron charge, $\hbar$ is the reduced Planck constant, μ_0 is the vacuum permeability, M_{s} is the saturation magnetization, and t_{F} is the thickness of the magnetic layer. The applied electric field E was estimated from the total resistance R of the bilayer device, the applied charge current I_{RF}, and the distance between the electrodes along the current-flow direction L: $E = I_{\mathrm{RF}} R/L$. Thus, our analysis does not rely on an estimation of the applied current distribution in the bilayer. Here, we also note that the composition of $Bi_{0.1}Sb_{0.9}$ used in the present wok is close to pure Sb, which is a highly conductive semimetal with the

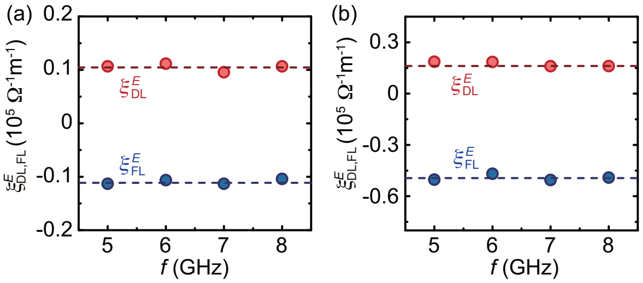

Supplementary Figure 6. The DL(FL)-SOT efficiency per electric field $\xi^E_{\mathrm{DL(FL)}}$ as a function of ST-FMR measurement frequency f for the (a) $Ni_{81}Fe_{19}$(6 nm)/$Bi_{0.1}Sb_{0.9}$ (10 nm) and (b) $Ni_{81}Fe_{19}$(6 nm)/$Bi_{0.1}Sb_{0.9}$ (40 nm) devices.

conductivity much higher than that of semimetal Bi and $Bi_{1-x}Sb_x$ in the semiconducting regime. In fact, the bulk resistivity of the $Bi_{0.1}Sb_{0.9}$ film used in this work is 350.3 $\mu\Omega$m, which is only less than two times larger than that of β-Ta (188.7 $\mu\Omega$m) [8]. The resistivity of the $Ni_{81}Fe_{19}$ layer has been determined to be 50.5 $\mu\Omega$m in the present work. Since the thickness of the $Bi_{0.1}Sb_{0.9}$ layer is much thicker than that of the $Ni_{81}Fe_{19}$ layer in most of the $Bi_{0.1}Sb_{0.9}$/$Ni_{81}Fe_{19}$ devices, a considerable current flows in the $Bi_{0.1}Sb_{0.9}$ layer in the ST-FMR measurement. For instance, in the $Ni_{81}Fe_{19}$(6 nm)/ $Bi_{0.1}Sb_{0.9}$(20 nm) bilayer, the ratio of the resistance of the $Ni_{81}Fe_{19}$ layer to that of the $Bi_{0.1}Sb_{0.9}$ layer is 0.42, showing that a large current flows in the $Bi_{0.1}Sb_{0.9}$ layer.

All the SOT efficiencies for the $Ni_{81}Fe_{19}$/$Bi_{0.1}Sb_{0.9}$ bilayers with different $Bi_{0.1}Sb_{0.9}$ thicknesses, shown in Fig. 3a in the main text, were estimated from the ST-FMR spectra measured at $f = 7$ GHz. We chose this frequency because the resonance peak of the spectra measured at $f = 7$ GHz appears in the middle of the applied field range, making the fitting to these spectra more reliable for extracting the symmetric and antisymmetric components. To validate this choice, we quantified the DL- and FL-SOT efficiencies for the $Ni_{81}Fe_{19}$(6 nm)/$Bi_{0.1}Sb_{0.9}$ (10 nm) and $Ni_{81}Fe_{19}$(6 nm)/$Bi_{0.1}Sb_{0.9}$ (40 nm) devices at different RF frequencies f ranging from 5 GHz to 8 GHz. The RF current at each f was individually calibrated. As demonstrated in Supplementary Fig. 6, both the DL- and FL-SOT efficiencies in these two devices are independent of the frequency. This result confirms the validity of the ST-FMR result shown in Fig. 3a in the main text.

In the second harmonic measurement, thermoelectric signals can be generated by Joule heating. In contrast, the Joule heating caused by current flow within the device does not

impact the ST-FMR analysis, as this contribution is irrelevant to the resonance signal. In the ST-FMR measurement, resonant heating from microwave absorption can give rise to thermoelectric signals with a symmetric Lorentzian shape at the FMR. We note that the resonant heating is generally less significant compared to the Joule heating [9]. We also note that the frequency dependence of the thermoelectric voltage due to the resonant heating is different from that of the ST-FMR voltage due to the DL SOT [9]. As shown in Supplementary Fig. 6, the DL-SOT efficiency extracted from the symmetric voltage for the $Ni_{81}Fe_{19}/Bi_{0.1}Sb_{0.9}$ bilayer is found to be independent of the RF frequency f, as expected. This result supports that the measured voltage is dominated by the ST-FMR, as the heating contribution will produce a frequency dependent result.

The torque efficiencies can also be quantified by measuring the ferromagnetic-layer thickness dependence of the V_s/V_a ratio. In this work, we avoid using this analysis. The reason for this is that this analysis is based on an assumption that both the DL and FL torque efficiencies are independent of the ferromagnetic layer thickness, and only the current flowing into the nonmagnetic layer accounts for the torque generation. In general, the validity of this assumption is not obvious; the assumptions are invalid when phenomena such as the anomalous spin-orbit torque, the interfacial spin dependent scattering, and the interfacial Rashba effect contribute to the spin-orbit torque.

Supplementary Note 3: Empirical fitting of the thickness dependent resistivity

We employed an empirical model to fit the thickness t dependence of the resistivity ρ for the $Bi_{0.1}Sb_{0.9}$ film at room temperature, shown in Fig. 3c in the main text. In this model, a difference in the conductivity of the bulk and at the surface is taken into account. Since the surface conductivity $\sigma_{\rm surf}$ is affected by electron reflection, $\sigma_{\rm surf}$ is expected to decay exponentially to the bulk value, as the distance from the surface increases. Thus, the position-dependent conductivity in the $Bi_{0.1}Sb_{0.9}$ film as a sum of the bulk and surface contributions is given by [10]

$$\sigma(z) = \frac{1}{\rho_{\rm BiSb}}\left[1 - \exp\left(\frac{-z}{\sigma_{\rm surf}\rho_{\rm BiSb}\lambda}\right)\right] + \sigma_{\rm surf}\exp\left(\frac{-z}{\sigma_{\rm surf}\rho_{\rm BiSb}\lambda}\right), \tag{S5}$$

where $\rho_{\rm BiSb}$ is the bulk resistivity, and λ is bulk mean free path. The product of $\sigma_{\rm surf}\rho_{\rm BiSb}\lambda$ describes the thickness over which $\sigma_{\rm surf}$ is effective. Through integrating $\sigma(z)$ over the $Bi_{0.1}Sb_{0.9}$ thickness from $z = 0$ to $z = t$, we have the final form of the thickness-dependent resistivity expressed as

$$\rho(t) = \frac{\rho_{\rm BiSb}}{1 + \left(\frac{\sigma_{\rm surf}\rho_{\rm BiSb}\lambda}{t}\right)(\sigma_{\rm surf}\rho_{\rm BiSb} - 1)\left[1 - \exp\left(\frac{-t}{\sigma_{\rm surf}\rho_{\rm BiSb}\lambda}\right)\right]}. \tag{S6}$$

Using equation (S6), the measured t dependence of ρ can be well fitted, as shown in Fig. 3c in the main text. This fitting result gives $\sigma_{\rm surf} = 2.0 \times 10^5\ \Omega^{-1}\mathrm{m}^{-1}$, $\rho_{\rm BiSb} = 350.3 \pm 5.9$ $\mu\Omega$cm, and $\lambda = (12.8 \pm 3.3)$ nm. Since this empirical model describes a scenario of the bulk transport in the same manner as in the heavy metal case, the good fitting to the data indicates that the bulk state dominates the conduction in the sputtered $Bi_{0.1}Sb_{0.9}$ films. This result shows that the thickness dependence of the resistivity, shown in Fig. 3c in the main text, arises from the surface reflection, and the bulk resistivity is unchanged by changing the thickness. This demonstrates that the bulk transport property is independent of the thickness of the $Bi_{0.1}Sb_{0.9}$ film.

Supplementary Note 4: Analysis of transport properties using a parallel conduction model

Supplementary Figure 7(a) shows temperature T dependence of the conductivity σ measured for a $Bi_{0.1}Sb_{0.9}$ single layer film with $t = 20$ nm at zero magnetic field. The result shows that σ is reduced by about 10% upon cooling from room temperature, indicating an insulating behaviour. This result illustrates the disordered nature of the semimetallic $Bi_{0.1}Sb_{0.9}$ film. A similar trend was observed for all the examined samples with t ranging from 6 to 70 nm as shown in Supplementary Fig. 7(b), consistent with that the transport property is independent of the thickness of the $Bi_{0.1}Sb_{0.9}$ film.

By applying a parallel conduction model involving metallic and hopping conduction [11, 12], we analyze the role of disorders in the sputtered $Bi_{0.1}Sb_{0.9}$ film. As shown in Supplementary Fig. 7(a), at $T > 150$ K, the insulating behaviour is well reproduced by a sum of two terms: a thermally activated hopping between neighboring grains, following the Arrhenius law $\sigma_1 = (\rho_1 e^{E_\mathrm{a}/kT})^{-1}$, and a nearly temperature independent metallic conductivity, $\sigma_\mathrm{M} = 1/\rho_\mathrm{M}$, which may either originate from the bulk or the surface states, or both. Here, k is the Boltzmann's constant, ρ_1 is the material-dependent constant, and E_a is the activation energy.

At relatively low temperatures, the hopping length of electrons becomes much longer than the grain size in the insulating channel and consequently the hopping between neighboring grains becomes forbidden. In this situation, the electrostatic disorder related to the presence of amorphous phases results in a co-tunnelling process, which falls in the Efros-Shklovskii (ES) variable range hopping (VRH) regime, i.e., $\sigma_2 \sim [e^{-(T_\mathrm{ES}/T)^{1/2}}]$, where T_ES is a characteristic temperature depending on the particular microscopic characteristics [13, 14]. This description can be proved by the T dependence of σ; the fit using the parallel conduction model starts to deviate from the data at $T = 150$ K (dashed red line in Supplementary Fig. 7(a)), unless the Arrhenius term is replaced by the ES VRH term for the insulating channel.

The averaged fitting parameters obtained from the thickness series are $E_\mathrm{a} = 56 \pm 2$ meV and $T_\mathrm{ES} = 1642 \pm 247$ K at relatively high and low temperatures, respectively. Thus, the ratio of σ_M/σ obtained from the parallel conduction model analysis turns to be $\sim$0.93 at room temperature, indicating that the conduction is dominated by the metallic channel.

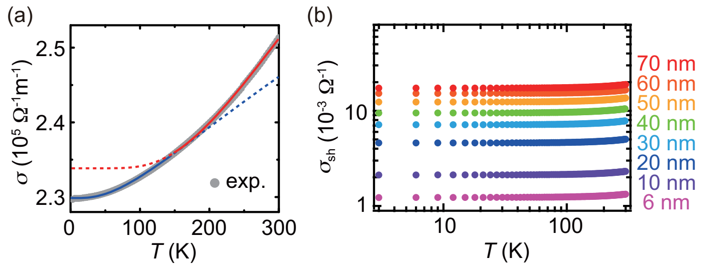

Supplementary Figure 7. (a) Temperature T dependence of the conductivity σ measured at zero magnetic field for the $Bi_{0.1}Sb_{0.9}$ film with the thickness of 20 nm. The solid circles are the experimental data. The temperature T dependence of σ can be reproduced by a parallel conduction model. At $T > 150$ K, the insulating behaviour obeys the Arrhenius law (red curve), whereas at $T \lesssim 150$ K, the decrease of σ with lowering T falls in the the Efros-Shklovskii (ES) variable range hopping (VRH) regime (blue curve). The dashed curves show the deviation from the fitting because of the crossover between the neighboring grain hopping and ES VRH mechanisms for the insulating component. (b) The sheet conductance $\sigma_{\rm sh}$ as a function of temperature T for the sputtered $Bi_{0.1}Sb_{0.9}$ films with different thicknesses ranging from 6 to 70 nm.

Supplementary Note 5: Thickness dependence of the torque efficiencies

In our analysis, the drift-diffusion model is used to describe the spin transport in the $Bi_{0.1}Sb_{0.9}$ layer induced by the spin Hall effect, while the spin transfer at the tunnelling interface is characterized by the spin mixing conductance. It is known that spin transport across a non-magnetic/magnetic interface is well characterized by the spin mixing conductance based on magnetoelectronic circuit theory [15]. In this model, the real part of the spin mixing conductance determines the spin transmission associated with the DL-torque generation and the imaginary part of the spin mixing conductance determines the reflection of spins exchange coupled to the interfacial magnetization associated with the FL-torque generation. For an energy barrier due to the creation of an ultrathin electron depletion layer, electron hopping in this region is allowed, which is dissimilar to the blocking of electron transport by an insulator. In this work, we have developed a theoretical model based on magnetoelectronic circuit theory to describe a change in the spin mixing conductance upon modulating the electron hopping. It is worth noting that the magnetoelectronic circuit theory is applicable to the analysis in ballistic, diffusive, and tunnelling spin transport regimes.

We discuss the resistivity change of the $Bi_{0.1}Sb_{0.9}$ layer induced by varying the thickness and its impact on the estimation of the spin diffusion length. As shown in Fig. 3c in the main text, the resistivity of the $Bi_{0.1}Sb_{0.9}$ layer decreases from 451.2 $\mu\Omega$m (t = 6 nm) to 370.5 $\mu\Omega$m (t = 70 nm) with increasing the thickness t. The 1.2 times change in the resistivity is moderate compared to the 4.5 times change in the resistivity of Pt in a thickness range from 1.2 nm to 15 nm [16]; since the thickness of the $Bi_{0.1}Sb_{0.9}$ layer was varied in the relatively thick regime, the influence of surface scattering on the resistivity is much weakened. Note that the resistivity change is even smaller, less than 7.5%, in the range from 20 mn to 70 nm as shown in Fig. 3c in the main text. The small change in the resistivity suggests that the spin transport in the $Ni_{81}Fe_{19}$/$Bi_{0.1}Sb_{0.9}$ bilayer is barely affected by the change in the thickness. Furthermore, a recent study suggests that the spin diffusion length is determined by the out-of-plane resistivity, rather than the in-plane resistivity when the spin current flows in the out-of-plane direction [17]. Thus, the spin diffusion length is independent of the thickness when the bulk resistivity does not depend on the thickness. We have confirmed that

the bulk resistivity in the $Bi_{0.1}Sb_{0.9}$ layer is independent of the thickness (see Supplementary Note 3), and therefore the spin diffusion length can be assumed to be a constant when varying the thickness.

To check the reliability of our analysis on the spin diffusion length λ_s, we fit the thickness dependence of the spin-orbit torque efficiency per electric field $\xi^E_{DL(FL)}$ in the thickness t ranging from 20 to 70 nm, where the change in the resistivity is less than 7.5%. From the fitting, we obtained $\sigma_s = (3.65 \pm 0.05) \times 10^5 (\hbar/2e)\ \Omega^{-1}m^{-1}$ and $\lambda_s = 25.6 \pm 1.1$ nm. These values are comparable to those obtained from the fitting to the data for the entire thickness range presented in the manuscript, $\sigma_s = (3.68 \pm 0.17) \times 10^5 (\hbar/2e)\ \Omega^{-1}m^{-1}$ and $\lambda_s = 24.8 \pm 1.0$ nm. This result indicates that the small resistivity change in the $Bi_{0.1}Sb_{0.9}$ layer in the $Ni_{81}Fe_{19}/Bi_{0.1}Sb_{0.9}$ devices does not affect the estimation of the spin diffusion length.

The spin diffusion length of the $Bi_{0.1}Sb_{0.9}$ obtained from the fitting is a reasonable value. Previous studies have shown that the spin diffusion length in Bi is larger than 15 nm [18, 19]. In our Sb rich alloy $Bi_{0.1}Sb_{0.9}$, the strength of the spin-orbit coupling is reduced relative to Bi [2], which suggests that the spin diffusion length in $Bi_{0.1}Sb_{0.9}$ can be longer than that in Bi. The relatively long spin diffusion length in Bi and $Bi_{0.1}Sb_{0.9}$ can be attributed to the small density of states at the Fermi level, compared to that of heavy metals. The spin diffusion length can be expressed as $\lambda_s = \sqrt{D\tau_s}$, where D is the diffusion coefficient and τ_s is the spin relaxation time. Since D is inversely proportional to the density of states at the Fermi level, D of Bi and $Bi_{0.1}Sb_{0.9}$ can be larger than that of heavy metals, such as Pt. We also note that $\tau_s = \tau_e/a$ in $Bi_{0.1}Sb_{0.9}$ can also be longer than that of heavy metals, where τ_e is the momentum relaxation time and a is the spin flip-probability. The reason for this is that τ_e is also inversely proportional to the density of states at the Fermi level.

Supplementary Note 6: Characterization of volume carrier densities

To estimate the carrier density, we measured the Hall effect for the $Bi_{0.1}Sb_{0.9}$ films. In Supplementary Fig. 8(a), we show the Hall resistance R_{xy} as a function of a perpendicular external field H at 2 K for $Bi_{0.1}Sb_{0.9}$ single layer films with t = 20, 40, and 60 nm. The positive slope in the H dependence of R_{xy} indicates that the charge transport is dominated by p-type carriers. Supplementary Figure 8(a) also shows that the slope decreases with increasing the thickness. This result suggests that the sheet carrier density depends on the thickness, which reflects the feature of bulk dominated conduction.

Due to the slight overlap between the conduction and valance bands in the semimetal, electrons with carrier population n_e spill over into the conduction band to leave an equal number of holes n_h in the valence band, giving $n = n_\mathrm{e} = n_\mathrm{h}$. This enables us to calculate the carrier density based on $n = (\mu_\mathrm{h} - \mu_\mathrm{e})/eR_\mathrm{H}(\mu_\mathrm{h} + \mu_\mathrm{e})$, where R_H is the Hall coefficient. μ_h and μ_e are the mobility of the hole and electron, respectively. Since the ratio $(\mu_\mathrm{h} - \mu_\mathrm{e})/(\mu_\mathrm{h} + \mu_\mathrm{e})$ is less than one, $n^* = 1/eR_\mathrm{H}$ can be regarded as an upper limit of the volume carrier density for the disordered $Bi_{0.1}Sb_{0.9}$ films. In Supplementary Fig. 8(b) we summarize $Bi_{0.1}Sb_{0.9}$-thickness, t, dependence of n^*. This result shows that n^* is a nearly constant value of around 0.6×10^{21} cm^{-3}, which supports that the film quality is independent of the thickness.

We also performed the Hall measurement at different temperatures T for a typical $Bi_{0.1}Sb_{0.9}$ single layer film with t = 50 nm. As shown in Supplementary Fig. 8(c), the slope of the H dependence of R_{xy} decreases with increasing T. In Supplementary Fig. 8(d), we show the extracted upper limit of the carrier density n^* as a function of T. From this result, we obtain the upper limit of the carrier density in the disordered $Bi_{0.1}Sb_{0.9}$ film as 1.16×10^{21} cm^{-3} at room temperature. Assuming the lower limit of the carrier density to be a calculated bulk carrier density of Sb at room temperature in the absence of structural defects [20], the carrier density of the disordered $Bi_{0.1}Sb_{0.9}$ film at room temperature is in the range of 0.1×10^{21} cm^{-3} $< n <$ 1.2×10^{21} cm^{-3}.

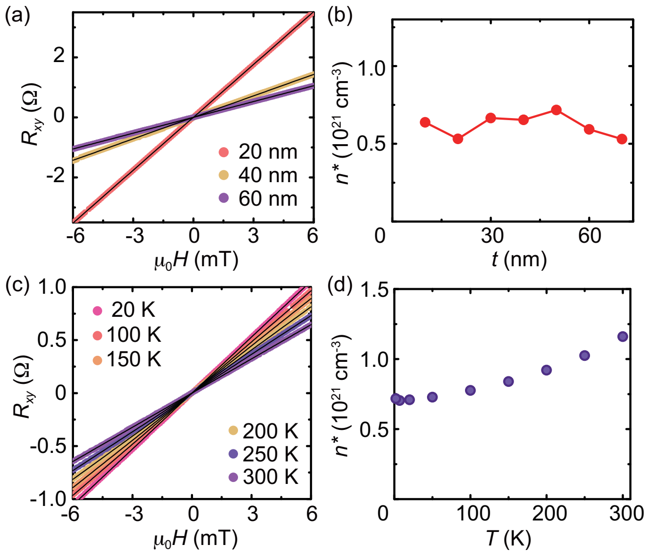

Supplementary Figure 8. (a) The Hall resistance R_{xy} measured with applying a perpendicular magnetic field H for the $Bi_{0.1}Sb_{0.9}$ films with different thicknesses t. (b) The upper limit of the volume carrier density n^* as a function of t at $T = 2$ K. (c) H dependence of R_{xy} for the $Bi_{0.1}Sb_{0.9}$ film with $T = 50$ nm at different temperatures. (d) The upper limit of n^* as a function of T.

Supplementary Note 7: Magnetic damping measurements

We estimate the effective spin mixing conductance by measuring the magnetic damping. In the $Ni_{81}Fe_{19}/Bi_{0.1}Sb_{0.9}$ bilayer at ferromagnetic resonance (FMR), the magnetization precession of the $Ni_{81}Fe_{19}$ layer pumps a spin current into the adjacent $Bi_{0.1}Sb_{0.9}$ layer by the spin pumping. This process results in the dissipation of the angular momentum from the $Ni_{81}Fe_{19}$ layer, leading to an enhancement of the effective Gilbert damping parameter α_G [21, 22].

Supplementary Figure 9(a) shows out-of-plane magnetic field angle ϕ_H dependence of the FMR peak-to-peak linewidth ΔH for the $Ni_{81}Fe_{19}$(12 nm)/$Bi_{0.1}Sb_{0.9}$(t nm) bilayer film and a $Ni_{81}Fe_{19}$(12 nm) single layer film, where the values of ΔH were extracted from FMR spectra measured with a microwave cavity. This result shows that ΔH is almost unchanged by attaching the $Bi_{0.1}Sb_{0.9}$ layer at $\phi_H = 90°$. In contrast, at $\phi_H = 0°$, the linewidth for the $Ni_{81}Fe_{19}/Bi_{0.1}Sb_{0.9}$ bilayer is notably larger than that for the $Ni_{81}Fe_{19}$ film. The clear difference in ΔH between $\phi_H = 0°$ and 90° is mainly caused by two-magnon scattering. It is known that apart from the enhancement of the damping due to the absorption of a pumped spin current, the linewidth broadening in bilayers can also be attributed to other two contributions; one is the inhomogeneous broadening due to the spread of anisotropy fields through the local variation of the resonance field [22, 23], and the other is two-magnon scattering induced by the surface/interface roughness and defects in the film volume [24–26]. Note that the two-magnon contribution disappears when the angle of magnetization relative to the film plane ϕ_H is larger than 45°, which ensures a distinct difference in linewidth broadening between the case of $\phi_H = 0°$ and 90°. Taking into account all these contributions [22, 26], we fitted the angular dependence of ΔH. The fitting result reveals that the two-magnon scattering contributes to the linewidth enhancement, whose magnitude remains relatively unchanged upon varying the thickness of the $Bi_{0.1}Sb_{0.9}$ layer at $\phi_H = 0°$ or 180°, likely pointing to an interface roughness related origin.

In Supplementary Fig. 9(b), we show the extracted values of α_G for the $Ni_{81}Fe_{19}/Bi_{0.1}Sb_{0.9}$ bilayer as a function of the thickness t of the $Bi_{0.1}Sb_{0.9}$ layer. This result shows that α_G is independent of t, demonstrating that the spin injection into the bulk of the $Bi_{0.1}Sb_{0.9}$ layer due to the spin pumping is negligible. The negligible spin pumping into the bulk states indicates negligible $\mathrm{Re}[G^{\uparrow\downarrow}]$, which is consistent with the small value of $\mathrm{Re}[G^{\uparrow\downarrow}]$ extracted

from the ST-FMR for the $Ni_{81}Fe_{19}/Bi_{0.1}Sb_{0.9}$ bilayer. We have also confirmed that the effective magnetization field $M_{\rm eff}$ of the $Ni_{81}Fe_{19}/Bi_{0.1}Sb_{0.9}$ bilayer is independent of the $Bi_{0.1}Sb_{0.9}$ thickness t, as shown in Supplementary Fig. 9(c).

Supplementary Figure 9(b) also shows that the enhancement of $\alpha_{\rm G}$, which is independent of t, relative to the damping α_0 of the reference $Ni_{81}Fe_{19}$ film is unremarkable. The thickness-independent damping enhancement can be attributed to an interfacial effect [27]. To make a direct comparison of our data with that reported in literatures, we calculated the effective spin mixing conductance $G_{\rm eff}^{\uparrow\downarrow}$ using the relation: $G_{\rm eff}^{\uparrow\downarrow} = e^2 M_{\rm s} t_{\rm FM}(\alpha_{\rm G} - \alpha_0)/g\mu_{\rm B}\hbar$ [28], where $M_{\rm s}$ and $t_{\rm FM}$ are the saturation magnetization and thickness of the $Ni_{81}Fe_{19}$ layer, respectively. The determined value of $G_{\rm eff}^{\uparrow\downarrow} = (9.7 \pm 2.5) \times 10^{13}\ \Omega^{-1}\ \mathrm{m}^{-2}$ for the $Ni_{81}Fe_{19}/Bi_{0.1}Sb_{0.9}$ bilayer is more than three times in magnitude smaller than $G_{\rm eff}^{\uparrow\downarrow} = 3.1 \times 10^{14}\ \Omega^{-1}\ \mathrm{m}^{-2}$ for a $Ni_{81}Fe_{19}$/Pt interface, indicating that the interfacial spin-orbit coupling effect is insignificant in the $Ni_{81}Fe_{19}/Bi_{0.1}Sb_{0.9}$ bilayer.

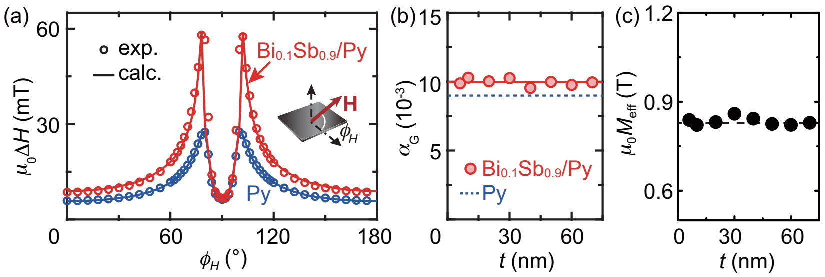

Supplementary Figure 9. (a) FMR peak-to-peak linewidth ΔH as a function of the out-of-plane angle ϕ_H of the static magnetic field H with respect to the film plane. The representative results (blue and red circles) are measurements on the $Ni_{81}Fe_{19}$(12 nm) and $Ni_{81}Fe_{19}$(12 nm)/$Bi_{0.1}Sb_{0.9}(t)$ films with $t = 60$ nm at room temperature. The solid lines are the theoretical calculation including the two magnon contribution, which well describes the experimental data. The inset shows the measurement geometry. (b) Thickness t dependence of the damping constants $\alpha_{\rm G}$ obtained by fitting the ϕ_H dependence of ΔH for the $Ni_{81}Fe_{19}$(12 nm)/$Bi_{0.1}Sb_{0.9}(t)$ films. The determined values of $\alpha_{\rm G}$ are nearly constant regardless of the thickness change, indicated by the solid line. The dashed line is the magnetic damping α_0 of the $Ni_{81}Fe_{19}$(12 nm) film. (c) Thickness t dependence of the effective demagnetization field $M_{\rm eff}$ for the $Ni_{81}Fe_{19}$(12 nm)/$Bi_{0.1}Sb_{0.9}(t)$ films.

Supplementary Note 8: Estimation of work function in binary alloy

For an A_xB_{1-x}-type binary alloy, the work function can be approximately expressed as [29]

$$\phi = x\phi_{\mathrm{A}} + (1-x)\phi_{\mathrm{B}} + x(1-x)\left[\frac{(\phi_{\mathrm{A}} - \phi_{\mathrm{B}})(\frac{\rho_{\mathrm{A}}(\varepsilon)}{\rho_{\mathrm{B}}(\varepsilon)} - 1)}{x\frac{\rho_{\mathrm{A}}(\varepsilon)}{\rho_{\mathrm{B}}(\varepsilon)} + (1-x)}\right] \tag{S7}$$

where ϕ_{A} and ϕ_{B} are the pure constituent work function of A and B, respectively; $\rho(\varepsilon)$ refers to the density of states at the Fermi level, which is proportional to the electronic specific heat constant $C_{\mathrm{e}} = (1/3)\pi^2\rho(\varepsilon)k_B^2T$. Here, k_B and T are the Boltzmann constant and temperature, respectively. Therefore, using equation (S7) with the parameters of the pure constituent work function determined in polycrystal [30] and the electronic specific heat constant, the $\phi_{\mathrm{Bi_{0.1}Sb_{0.9}}}$ and $\phi_{\mathrm{Co_{72}Tb_{28}}}$ can be roughly estimated as given in the main text.

One of the most widely studied Bi-based alloys in spintronics is Bi_2Se_3. For Bi_2Se_3/FM (FM = Co, CoFeB, Permalloy, and Fe) systems, the Fermi level of Bi_2Se_3 lies below that of all the FMs; the work function of Bi_2Se_3 is 5.6 eV, while that of Co, CoFeB, Permalloy, and Fe are 5.0, 4.8, 4.83, and 4.5 eV, respectively. In this case, the band of Bi_2Se_3 near the interface bends downward, creating an electron accumulation region rather than an electron depletion region. Thus, no energy barrier is expected in the Bi_2Se_3/FM (FM = Co, CoFeB, Permalloy, and Fe) heterojunctions. The SOT generation by Bi-rich BiSb alloys has been studied using in $Bi_{0.9}Sb_{0.1}$/MnGa bilayers [31]. Since the work functions of MnGa and $Bi_{0.9}Sb_{0.1}$ are estimated to be around 4.11 and 4.42 eV, respectively, using equation (S7), no energy barrier is expected in this system.

Since charge transfer in a heterojunction is a general effect, the effect of an inherent energy barrier on the spin transport, and its consequences on the generation of the spin-orbit torques should be taken into account in future studies, especially when the work function of a lower carrier bulk spin source is smaller than that of a magnetic layer.

Supplementary Note 9: Spin-mixing conductance: theoretical model

To study the spin-mixing conductance, we consider a toy model representing the interface of a metal-magnet structure. We model the metallic heterostructure with a tunnelling region at the interface, see Fig. 4 in the main text, by a tight-binding Hamiltonian defined in a semi-infinite 3D cubic lattice,

$$H = t_0 \sum_{\langle ij \rangle} \hat{c}^{\dagger}_{\boldsymbol{r}_i} \hat{c}_{\boldsymbol{r}_j} + J \sum_{\boldsymbol{r}_i} \boldsymbol{m} \cdot \hat{\boldsymbol{s}}_{\boldsymbol{r}_i} + t_m \sum_{\langle ij \rangle} \hat{c}^{\dagger}_{\boldsymbol{x}_i} \hat{c}_{\boldsymbol{x}_j}, \tag{S8}$$

where $\boldsymbol{r}_i$ represents the atomic sites at the semimetalic layer (a semi-infinite 3D bulk layer), while $\boldsymbol{x}_i$ denotes the lattice positions at the 2D ferromagnetic metal layer, inserted in our model as a boundary condition. The hopping integrals are t_0 and t_m at the semimetal and magnetic parts, respectively. The tunnelling and hopping between two layers, via the energy barrier induced by the depletion region, are modelled by a spin-dependent exchange coupling between spin of conduction electrons at semimetal layer and local magnetic moments at magnetic metal layer J, while the spin-independent tunnelling is encoded in the hoping mismatch parameter $\delta t = t_0 - t_m$. According to the scattering formalism for spin-pumping [32], the real and imaginary parts of the spin-mixing conductance are given by

$$\mathrm{Re}\left[G^{\uparrow\downarrow}\right] = \frac{e^2 \mathcal{A}}{h\pi^2} \int |\Delta S|^2 dk_y dk_z, \tag{S9}$$

$$\mathrm{Im}\left[G^{\uparrow\downarrow}\right] = \frac{e^2 \mathcal{A}}{h\pi^2} \int \mathrm{Im}\left[S_0^* \Delta S\right] dk_y dk_z. \tag{S10}$$

Up to linear order in $\boldsymbol{m}$, the scattering matrix is $S = S_0 + S_w \sigma_0 + \Delta S (\boldsymbol{m} \cdot \boldsymbol{\sigma})$, with $\boldsymbol{\sigma}$ the Pauli matrices and σ_0 the identity matrix. The transverse momenta are k_y and k_z, $\mathcal{A}$ the interface cross section, h the Planck constant, and e the elementary electron charge. We evaluate the scattering matrix by applying a standard approach, developed in Refs. [33–35], and compute the spin-mixing conductance as a function of two dimensionless parameters $\delta t/t_0 = 1 - t_m/t_0$ and J/t_0. The result for the real- and imaginary-part is plotted at Fig. 4 in the main text.

Supplementary Note 10: Ordinary Nernst effect in second harmonic signals

Owing to the low thermal conductivity of $Bi_{0.1}Sb_{0.9}$, the ordinary Nernst effect (ONE) can produce a large second harmonic Hall signal $R_{\rm ONE}$ in the presence of an out-of-plane temperature gradient ΔT [36]. This thermal contribution scales linearly with the in-plane longitudinal component of the external magnetic field H. In our measurement geometry (see Fig. 5a in the main text), the rotation of H in the xz plane changes the component of the field along the x axis. Thus, the second harmonic Hall signal $R_{2\omega}^{\rm ONE}$ originating from the ONE due to an alternating current $I = I_0 \sin \omega t$ is written as $R_{2\omega}^{\rm ONE} = (\alpha N \Delta T / I_0)\mu_0 H \sin\theta_H$. Here, θ_H is the angle of the external magnetic field relative to the film normal, α is the geometrical factor, and N is the ordinary Nernst coefficient. By taking into account the thermal contribution, the second harmonic Hall signal $\tilde{R}_{2\omega}$ due to an alternating current $I = I_0 \sin \omega t$ is expressed as [36, 37]

$$\tilde{R}_{2\omega} = \frac{\alpha N \Delta T \mu_0 H}{I_0}\sin\theta_H - \frac{1}{2}R_{\rm AHE}\frac{H'_{\rm DL}}{H + H_{\rm K}}\sin\theta_M - \frac{A\alpha\Delta T}{I_0}\sin\theta_M. \quad \text{(S11)}$$

The first term is the signal due to the ONE. The second term is the signal generated by the current-induced DL effective field $H_{\rm DL}$, where $H'_{\rm DL} = H_{\rm DL}(1 - 4\xi^2)$. Here, $\xi = R_{\rm PHE}/R_{\rm AHE}$, $R_{\rm AHE}$ is the anomalous Hall coefficient, and $R_{\rm PHE}$ is the planar Hall resistance. $H_{\rm K}$ is the anisotropy field and θ_M is the out-of-plane angle of the net magnetization. The third term represents the contribution from the anomalous Nernst effect (ANE), whose magnitude is related to the coefficient A for the ANE.

In Supplementary Fig. 10, we plot measured values of $\tilde{R}_{2\omega}$ as a function of H at various θ_H. The ONE contribution $R_{2\omega}^{\rm ONE}$ in the measured values of $\tilde{R}_{2\omega}$ can be extracted by fitting the H dependence of the measured values of $\tilde{R}_{2\omega}/\sin(\theta_M - \pi)$ at each θ_H using equation (S11). In Fig. 5b in the main text, we plot θ_M dependence of $R_{2\omega} = \tilde{R}_{2\omega} - R_{2\omega}^{\rm ONE}$, where the ONE contribution is subtracted from the measured second harmonic signals. We extracted $H'_{\rm DL}$ by fitting the measured $1/(H + H_{\rm K})$ dependence of $R_{2\omega}$ using $dR_{2\omega}/d(\sin\theta_M) = -R_{2\omega}^{\rm ANE} - (1/2)R_{\rm AHE}H'_{\rm DL}/(H + H_{\rm K})$, where $R_{2\omega}^{\rm ANE} = A\alpha\Delta T/I_0$ (see equation (S11)).

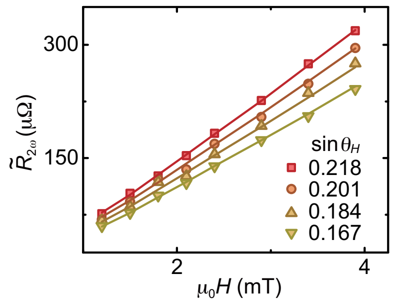

Supplementary Figure 10. Second harmonic Hall resistance $\tilde{R}_{2\omega}$ as a function of the external magnetic field H for the representative $Co_{72}Tb_{28}$(6 nm)/$Bi_{0.1}Sb_{0.9}$(10 nm) sample measured at room temperature, when the angle θ_H of the field is fixed at certain values. Solid lines represent the fitting to the data using equations (S11).

Supplementary Note 11: Temperature dependence of saturation magnetization and planar Hall resistance

In Supplementary Fig. 11(a), we show temperature T dependence of the saturation magnetization M_s for $Co_{72}Tb_{28}$(6 nm)/$Bi_{0.1}Sb_{0.9}$(10 nm) film measured using a vibrating sample magnetometer. This result shows that M_s increases with decreasing T. The temperature-dependent change of M_s has been taken into account in the estimation of the T dependence of the DL-torque efficiency shown in Fig. 5c in the main text.

We also measured T dependence of $\xi = R_{\mathrm{PHE}}/R_{\mathrm{AHE}}$, which relates H'_{DL} and H_{DL} as $H_{\mathrm{DL}} = H'_{\mathrm{DL}}/(1-4\xi^2)$ (see equation (S11)). To evaluate the planar Hall resistance R_{PHE}, we measured in-plane magnetic field angle dependence of the Hall resistance R_{xy} for the $Co_{72}Tb_{28}$(6 nm)/$Bi_{0.1}Sb_{0.9}$(10 nm) device, as shown in Supplementary Fig. 11(b). The measurements were performed by rotating the external magnetic field $\mu_0 H = 9$ T in the film plane. The change of the in-plane field angle θ relative to the applied current direction leads to the variation of R_{xy} as

$$R_{xy} = R_{\mathrm{PHE}} \sin(2\theta) + R_0 \sin(\theta + \theta_0), \tag{S12}$$

where the first term corresponds to the planar Hall contribution, and the second term is from the anomalous Hall contribution due to the small out-of-plane tilting of the sample. θ_0 is the offset angle from the measurement setup. By fitting the data of the planar Hall measurements using equations (S12), R_{PHE} can be extracted. Using the obtained temperature dependence of the anomalous Hall resistance R_{AHE}, we evaluate the ratio $\xi = R_{\mathrm{PHE}}/R_{\mathrm{AHE}}$ as a function of temperature shown in Supplementary Fig. 11(c). This result shows that the largest value of $1/(1-4\xi^2)$ is 1.09 at 20 K, indicating that the difference between H_{DL} and H'_{DL} is less than 9% in the entire temperature range.

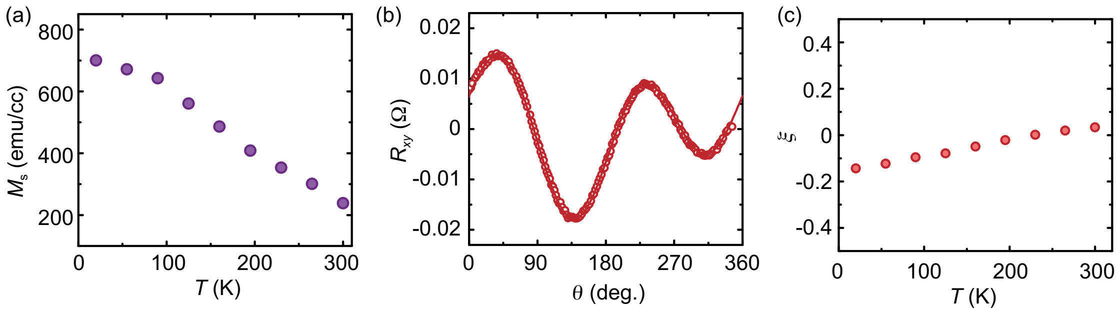

Supplementary Figure 11. (a) Temperature T dependence of the saturation magnetization M_s for the $Co_{72}Tb_{28}$(6 nm)/$Bi_{0.1}Sb_{0.9}$(10 nm) film. (b) In-plane magnetic field angle θ dependence of the Hall resistance R_{xy} for the $Co_{72}Tb_{28}$(6 nm)/$Bi_{0.1}Sb_{0.9}$(10 nm) device measured with an external magnetic filed of 9 T. θ is the magnetic field angle relative to the applied current direction. (c) T dependence of $\xi = R_\mathrm{PHE}/R_\mathrm{AHE}$ for the $Co_{72}Tb_{28}$(6 nm)/$Bi_{0.1}Sb_{0.9}$(10 nm) film, where R_AHE and R_PHE are the the anomalous Hall coefficient and planar Hall resistance, respectively.

Supplementary Note 12: Current-induced room temperature magnetization switching

We demonstrate current-induced magnetization switching using the SOT originating from the bulk of the topological semimetal. Supplementary Fig. 12(a) shows the Hall resistance R_{H} for the $Co_{72}Tb_{28}$(6 nm)/$Bi_{0.1}Sb_{0.9}$(10 nm) as a function of an applied DC current density j_{DC}, where an in-plane magnetic field is applied along the DC current direction. This result shows that the switching polarity converts from clockwise to counterclockwise rotation when the in-plane magnetic field direction is reversed. This result is consistent with the prediction of the current-induced magnetization switching by the SOT, suggesting that the joule heating plays a minor role during the switching [38, 39]. Here, we estimate the strength of the perpendicular magnetic anisotropy in the $Co_{72}Tb_{28}$/$Bi_{0.1}Sb_{0.9}$ device from the first harmonic measurement by rotating an out-of-plane external field $\mu_0 H = 1.5$ T. In Supplementary Fig. 12(b), we plot magnetic-field angle θ_H dependence of R_ω (red circles) and net-magnetization-angle θ_M dependence of R_ω (blue circles), where θ_M was estimated using $\theta_M = \arccos(R_\omega/R_{\mathrm{AHE}}) - \pi$. From this result we plot $H\sin(\theta_H - \theta_M)$ dependence of $(1/2)\sin(2\theta_M)$ in Supplementary Fig. 12(c). By fitting this result using the equilibrium equation [40], $(1/2)\sin(2\theta_M) = H\sin(\theta_H - \theta_M)/H_{\mathrm{K}}$, we obtain a strong perpendicular magnetic anisotropy field $\mu_0 H_{\mathrm{K}} = 1.34$ T, which is consistent with the large coercive field observed in the anomalous Hall resistance as a function of out-of plane magnetic field shown in Supplementary Fig. 12(d). This result indicates that the SOT enhanced by the energy barrier engineering allows to switch the magnetization with strong the perpendicular magnetic anisotropy.

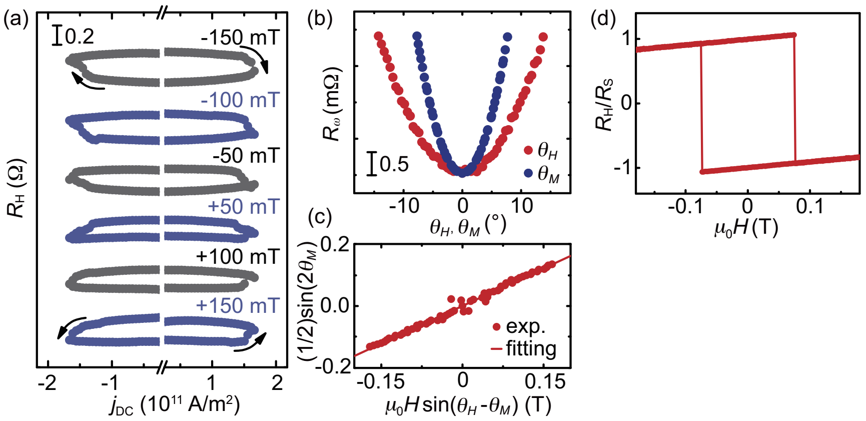

Supplementary Figure 12. (a) $R_{\mathrm{H}} - j_{\mathrm{DC}}$ loops obtained under various in-plane assisting magnetic fields for the $Co_{72}Tb_{28}$(6 nm)/$Bi_{0.1}Sb_{0.9}$(10 nm) bilayer, where R_{H} and j_{DC} are the Hall resistance and the applied DC current density in the $Bi_{0.1}Sb_{0.9}$ layer, respectively. The magnitude of the in-plane magnetic field ranges from -150 to $+150$ mT. (b) The first harmonic signal R_ω as a function of θ_H and θ_M measured with $\mu_0 H = 1.5$ T for the $Co_{72}Tb_{28}$(6 nm)/$Bi_{0.1}Sb_{0.9}$(10 nm) bilayer, where θ_H and θ_M are the angle of the external field and net magnetization with respect to the film normal, respectively. (c) The relation between $(1/2)\sin(2\theta_M)$ and $\mu_0 H \sin(\theta_H - \theta_M)$. The solid line is a linear fit to the data. (d) Normalized Hall resistance R_{H} of the $Co_{72}Tb_{28}$(6 nm)/$Bi_{0.1}Sb_{0.9}$(10 nm) bilayer as a function of the perpendicular magnetic field.

Supplementary Note 13: Characterization of field-like torque by second harmonic measurement

We performed the current induced in-plane effective field characterization on the $Co_{72}Tb_{28}$(6 nm)/$Bi_{0.1}Sb_{0.9}$(10 nm) film at room temperature. The in-plane effective field is the sum of the field-like effective field and the Oersted field: $H_{\mathrm{FL}} + H_{\mathrm{Oe}}$. In Supplementary Figs. 13(a) and 13(b), we show the first and second harmonic Hall resistances, R_ω and $R_{2\omega}$, measured by rotating the external magnetic field in the yz plane (see Fig. 5a in the main text). As shown in Supplementary Fig. 13(a), the change in R_ω as a function of the angle between directions of the film normal and magnetization θ_M opens upward, which is consistent with the picture that the net magnetization is dominated by Tb in the $Co_{72}Tb_{28}$ layer. In this measurement geometry, the second harmonic signal $R_{2\omega}$ has the form: $R_{2\omega} = (1/2)R_{\mathrm{AHE}} \sin\theta_M (H_{\mathrm{FL}} + H_{\mathrm{Oe}})/(H + H_{\mathrm{K}})$. In Supplementary Fig. 13(b), we show the θ_M dependence of $R_{2\omega}$ at different magnetic field strengths. Supplementary Figure 13(b) shows that $R_{2\omega}$ is almost independent of θ_M, indicating that the in-plane effective field, $H_{\mathrm{FL}} + H_{\mathrm{Oe}}$, barely contributes to the detected signals. Using the result shown in Supplementary Fig. 13(b), we plot $dR_{2\omega}/d(\sin\theta_M)$ as a function of $1/\mu_0(H + H_K)$ in Supplementary Fig. 13(c). From this result, we obtain $\mu_0 H_{\mathrm{FL}} + \mu_0 H_{\mathrm{Oe}} = 0.002 \pm 0.021$ mT, indicating that the sum of the field-like effective field and the Oersted field is below the measurement accuracy. Even though this makes it challenging to determine H_{FL}, we roughly estimate the FL-SOT efficiency assuming $H_{\mathrm{FL}} + H_{\mathrm{Oe}} = 0$ and calculating H_{Oe} based on the Ampère's law. We obtain $\xi^E_{\mathrm{FL}} \sim -0.1 \times 10^5\ \Omega^{-1}\ \mathrm{m}^{-1}$, which is clearly smaller than the DL-SOT efficiency $\xi^E_{\mathrm{DL}} \sim 0.5 \times 10^5\ \Omega^{-1}\ \mathrm{m}^{-1}$, shown in Fig. 3a in the main text.

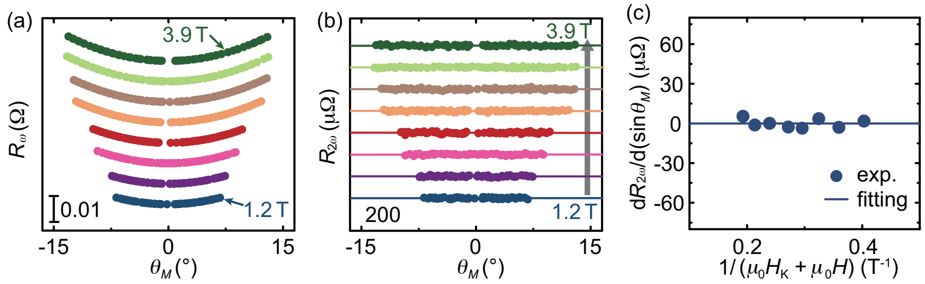

Supplementary Figure 13. Harmonic Hall measurements on the $Co_{72}Tb_{28}$(6 nm)/$Bi_{0.1}Sb_{0.9}$(10 nm) bilayer at room temperature by rotating an external magnetic field in the yz plane. (a) First R_ω, and (b) second $R_{2\omega}$ harmonic signals as a function of the magnetization angle θ_M. The signals were recorded simultaneously under different external magnetic fields from 1.2 to 3.9 T. (c) The current induced in-plane effective field signals as a function of the inverse field $1/(\mu_0 H + \mu_0 H_K)$.

Supplementary Note 14: Characterization of topological surface states

The generation of SOTs and magnetization switching in ferromagnet/topological-insulator heterostructures are generally attributed to topological surface states (TSSs) [31, 41–45]. While the thickness dependence of the $Bi_{0.1}Sb_{0.9}$ resistivity and the SOTs in our magnetic-metal/$Bi_{0.1}Sb_{0.9}$ bilayers suggest a pure bulk nature, one may wonder whether the TSSs contribute to the electron transport in our disordered $Bi_{0.1}Sb_{0.9}$ films.

To investigate the surface transport in the $Bi_{0.1}Sb_{0.9}$ film, we measured the magneto-conductance (MC) with applying a perpendicular magnetic field. In the quantum diffusive regime, a magnetic field perpendicular to the closed electron path will break its time reversal symmetry and destroy the quantum interference, manifested by the observation of the MC [46, 47]. Supplementary Figure. 14(a) shows the conductance change $\Delta\sigma_{\mathrm{sh}}(H) = \sigma_{\mathrm{sh}}(H) - \sigma_{\mathrm{sh}}(0)$ as a function of the perpendicular magnetic field H for $Bi_{0.1}Sb_{0.9}$ films with various thicknesses t at 2 K. The observed sharp cusp with negative MC is attributed to the weak antilocalization (WAL) effect, correlated to the destructive interference effect that suppresses the backscattering and gives rise to the enhanced conductance at zero field due to the strong spin-orbit coupling. The low field MC can be quantitively described by the simplified Hikami-Larkin-Nagaoka (HLN) formula [46].

$$\Delta\sigma_{\mathrm{sh}} = \tilde{\alpha}\frac{e^2}{2\pi^2\hbar}\left[\ln\left(\frac{B_\phi}{\mu_0 H}\right) - \psi\left(\frac{1}{2} + \frac{B_\phi}{\mu_0 H}\right)\right], \tag{S13}$$

where ψ is the digamma function and $B_\phi = \hbar/(4eL_\phi^2)$ is the phase coherence field. L_ϕ is the phase coherence length. The prefactor $\tilde{\alpha}$ determines the number of independent coherent channels with $\tilde{\alpha} = 0.5$ for a single transport channel. In our samples, due to the semimetallic nature with strong spin-orbit coupling, the bulk state should be in the WAL regime and give $\tilde{\alpha} = 0.5$, just like the surface and coupled surface-bulk states. Transport from multiple channels could be also available for topological insulators, such as decoupled surface and bulk, or independent top and bottom surface states, leading to $\tilde{\alpha} = 1$. As shown in Supplementary Fig. 14(b), we obtain $\tilde{\alpha} \approx 0.6$ for the $Bi_{0.1}Sb_{0.9}$ films from the fitting results shown in Supplementary Fig. 14(a)

Although it is hard to distinguish between the bulk and surface contributions based on the WAL results alone [48], $\tilde{\alpha} \approx 0.6$ for the $Bi_{0.1}Sb_{0.9}$ films is more likely in accordance with the intermediate coupling between the surface and bulk, and may be regarded as sig-

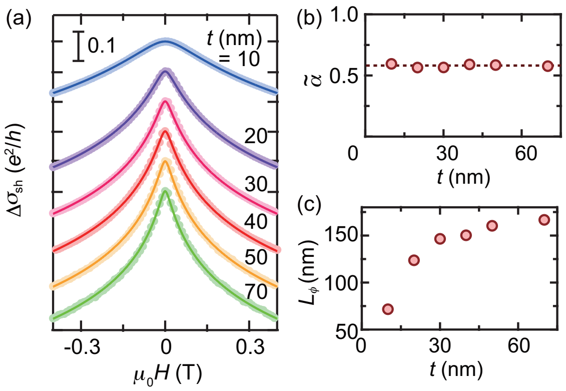

Supplementary Figure 14. (a) Magneto-conductance measurements on $Bi_{0.1}Sb_{0.9}$ films in the low field regime taken at $T = 2$ K, and the solid lines are their fits to the Hikami-Larkin-Nagaoka (HLN) equation. The prefactor $\tilde{\alpha}$ (b) and the coherence length L_ϕ (c) extracted from the HLN fitting plotted as a function of the $Bi_{0.1}Sb_{0.9}$ thickness t. Here, $\tilde{\alpha}$ defines the effective number of two-dimensional conducting channels, and the dashed line is a guide to the eye.

natures for the presence of the surface contribution. Besides, we find that L_ϕ for all the samples is remarkably larger than their thicknesses as shown in Supplementary Fig. 14(c), which proves that our system is effectively 2D at 2 K. The enhanced L_ϕ with the increase of t unambiguously verifies the conducting bulk, consistent with the results of the transport measurements. To provide further evidences, we performed WAL measurements at various temperatures as shown in Supplementary Fig. 15(a). As shown in Supplementary Fig. 15(b), as the temperature increases to 9 K, $\tilde{\alpha}$ decays to $\sim$0.5, which corresponds to a single transport channel. This result suggests that when the temperature is increased, the bulk becomes more conductive, which gives rise to a fully coupled channel composed of surface and bulk states. These results indicate the presence of additional channels related to the TSSs in the $Bi_{0.1}Sb_{0.9}$ film. Thus, the lack of surface contributions in our SOT observation given in the main text can be attributed to either the destruction of the TSSs by the interface hybridization [49, 50] or a minuscule density of spin polarization induced by a charge current in the TSSs [51].

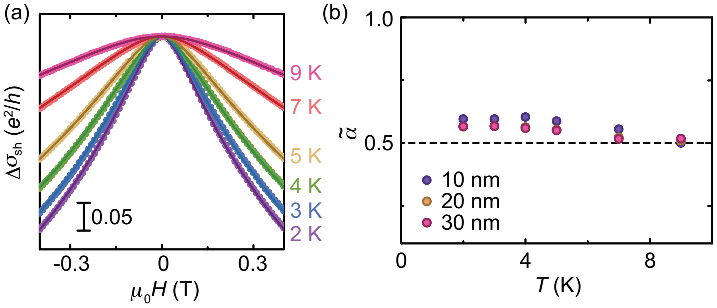

Supplementary Figure 15. (a) Magneto-conductance measurements on a $Bi_{0.1}Sb_{0.9}$ film with $t =$ 10 nm at various temperatures. (b) Temperature T dependence of the extracted prefactor $\tilde{\alpha}$. The dashed line is a guide to the eye laying on the value of the $\tilde{\alpha}$ that corresponds to a single transport channel.

Supplementary References

[S1] Hasan, M. Z. & Kane, C. L. Colloquium: topological insulators. *Rev. Mod. Phys.* **82**, 3045 (2010).

[S2] Şahin, C. & Flatté, M. E. Tunable giant spin Hall conductivities in a strong spin-orbit semimetal: $Bi_{1-x}Sb_x$. *Phys. Rev. Lett.* **114**, 107201 (2015).

[S3] Liu, L., Moriyama, T., Ralph, D. & Buhrman, R. Spin-torque ferromagnetic resonance induced by the spin Hall effect. *Phys. Rev. Lett.* **106**, 036601 (2011).

[S4] Fang, D. *et al.* Spin-orbit-driven ferromagnetic resonance. *Nat. Nanotechnol.* **6**, 413–417 (2011).

[S5] Gao, T. *et al.* Intrinsic spin-orbit torque arising from the Berry curvature in a metallic-magnet/Cu-oxide interface. *Phys. Rev. Lett.* **121**, 017202 (2018).

[S6] Spicer, T. M. *et al.* Spatial mapping of torques within a spin Hall nano-oscillator. *Phys. Rev. B* **98**, 214438 (2018).

[S7] Tshitoyan, V. *et al.* Electrical manipulation of ferromagnetic NiFe by antiferromagnetic IrMn. *Phys. Rev. B* **92**, 214406 (2015).

[S8] Liu, L. *et al.* Spin-torque switching with the giant spin Hall effect of tantalum. *Science* **336**, 555–558 (2012).

[S9] Karimeddiny, S., Mittelstaedt, J. A., Buhrman, R. A. & Ralph, D. C. Transverse and longitudinal spin-torque ferromagnetic resonance for improved measurement of spin-orbit torque. *Phys. Rev. Appl.* **14**, 024024 (2020).

[S10] Berger, A. J. *et al.* Determination of the spin Hall effect and the spin diffusion length of Pt from self-consistent fitting of damping enhancement and inverse spin-orbit torque measurements. *Phys. Rev. B* **98**, 024402 (2018).

[S11] Fritzsche, H. Electrical properties of germanium semiconductors at low temperatures. *Phys, Rev.* **99**, 406 (1955).

[S12] Gao, B., Gehring, P., Burghard, M. & Kern, K. Gate-controlled linear magnetoresistance in thin Bi_2Se_3 sheets. *Appl. Phys. Lett.* **100**, 212402 (2012).

[S13] Efros, A. & Shklovskii, B. I. Coulomb gap and low temperature conductivity of disordered systems. *J. Phys. C* **8**, L49 (1975).

[S14] Beloborodov, I., Lopatin, A., Vinokur, V. & Efetov, K. Granular electronic systems. *Rev. Mod. Phys.* **79**, 469 (2007).

[S15] Tserkovnyak, Y., Brataas, A., Bauer, G. E. & Halperin, B. I. Nonlocal magnetization dynamics in ferromagnetic heterostructures. *Rev. Mod. Phys.* **77**, 1375 (2005).

[S16] Nguyen, M.-H., Ralph, D. C. & Buhrman, R. A. Spin torque study of the spin Hall conductivity and spin diffusion length in platinum thin films with varying resistivity. *Phys. Rev. Lett.* **116**, 126601 (2016).

[S17] Gonzalez-Fuentes, C. *et al.* Spin diffusion length associated with out-of-plane conductivity of Pt in spin pumping experiments. *Phys. Rev. B* **103**, 224403 (2021).

[S18] Emoto, H. *et al.* Transport and spin conversion of multicarriers in semimetal bismuth. *Phys. Rev. B* **93**, 174428 (2016).

[S19] Cheng, J. *et al.* Coherent picture on the pure spin transport between Ag/Bi and ferromagnets. *Phys. Rev. Lett.* **129**, 097203 (2022).

[S20] Heremans, J., Thrush, C., Lin, Y.-M., Cronin, S. & Dresselhaus, M. Transport properties of antimony nanowires. *Phys. Rev. B* **63**, 085406 (2001).

[S21] Tserkovnyak, Y., Brataas, A. & Bauer, G. E. W. Enhanced Gilbert damping in thin ferromagnetic films. *Phys. Rev. Lett.* **88**, 117601 (2002).

[S22] Mizukami, S., Ando, Y. & Miyazaki, T. Effect of spin diffusion on Gilbert damping for a very thin permalloy layer in Cu/permalloy/Cu/Pt films. *Phys. Rev. B* **66**, 104413 (2002).

[S23] Mizukami, S., Ando, Y. & Miyazaki, T. The study on ferromagnetic resonance linewidth for NM/80NiFe/NM (NM=Cu, Ta, Pd and Pt) films. *Jpn. J. Appl. Phys.* **40**, 580 (2001).

[S24] Arias, R. & Mills, D. Extrinsic contributions to the ferromagnetic resonance response of ultrathin films. *Phys. Rev. B* **60**, 7395 (1999).

[S25] Landeros, P., Arias, R. E. & Mills, D. Two magnon scattering in ultrathin ferromagnets: The case where the magnetization is out of plane. *Phys. Rev. B* **77**, 214405 (2008).

[S26] Lindner, J. *et al.* Two-magnon damping in thin films in case of canted magnetization: Theory versus experiment. *Phys. Rev. B* **80**, 224421 (2009).

[S27] Rojas-Sánchez, J.-C. *et al.* Spin pumping and inverse spin Hall effect in platinum: the essential role of spin-memory loss at metallic interfaces. *Phys. Rev. Lett.* **112**, 106602 (2014).

[S28] Liu, Y., Yuan, Z., Wesselink, R. J., Starikov, A. A. & Kelly, P. J. Interface enhancement of Gilbert damping from first principles. *Phys. Rev. Lett.* **113**, 207202 (2014).

[S29] Gelatt Jr, C. & Ehrenreich, H. Charge transfer in alloys: AgAu. *Phys. Rev. B* **10**, 398 (1974).

[S30] Tran, R. *et al.* Anisotropic work function of elemental crystals. *Surf. Sci.* **687**, 48–55 (2019).

[S31] Khang, N. H. D., Ueda, Y. & Hai, P. N. A conductive topological insulator with large spin Hall effect for ultralow power spin–orbit torque switching. *Mat. Mater.* **17**, 808–813 (2018).

[S32] Tserkovnyak, Y., Brataas, A. & Bauer, G. E. W. Enhanced gilbert damping in thin ferromagnetic films. *Phys. Rev. Lett.* **88**, 117601 (2002).

[S33] Cheng, R., Xiao, J., Niu, Q. & Brataas, A. Spin pumping and spin-transfer torques in antiferromagnets. *Phys. Rev. Lett.* **113**, 057601 (2014).

[S34] Cheng, R. Aspects of antiferromagnetic spintronics Ph.D. thesis (2014).

[S35] Cogulu, E. *et al.* Quantifying spin-orbit torques in antiferromagnet–heavy-metal heterostructures. *Phys. Rev. Lett.* **128**, 247204 (2022).

[S36] Roschewsky, N. *et al.* Spin-orbit torque and Nernst effect in Bi-Sb/Co heterostructures. *Phys. Rev. B* **99**, 195103 (2019).

[S37] Yang, H., Chen, H., Tang, M., Hu, S. & Qiu, X. Characterization of spin-orbit torque and thermoelectric effects via coherent magnetization rotation. *Phys. Rev. B* **102**, 024427 (2020).

[S38] Finley, J. & Liu, L. Spin-orbit-torque efficiency in compensated ferrimagnetic cobalt-terbium alloys. *Phys. Rev. Applied* **6**, 054001 (2016).

[S39] Pham, T. H. *et al.* Thermal contribution to the spin-orbit torque in metallic-ferrimagnetic systems. *Phys. Rev. Applied* **9**, 064032 (2018).

[S40] He, P. *et al.* Quadratic scaling of intrinsic Gilbert damping with spin-orbital coupling in $L1_0$ FePdPt films: Experiments and *ab initio* calculations. *Phys. Rev. Lett.* **110**, 077203 (2013).

[S41] Mellnik, A. *et al.* Spin-transfer torque generated by a topological insulator. *Nature* **511**, 449–451 (2014).

[S42] Fan, Y. *et al.* Magnetization switching through giant spin–orbit torque in a magnetically doped topological insulator heterostructure. *Nat. Mater.* **13**, 699–704 (2014).

[S43] Han, J. *et al.* Room-temperature spin-orbit torque switching induced by a topological insulator. *Phys. Rev. Lett.* **119**, 077702 (2017).

[S44] Mahendra, D. *et al.* Room-temperature high spin–orbit torque due to quantum confinement in sputtered $Bi_xSe_{(1\text{-}x)}$ films. *Nat. Mater.* **17**, 800 (2018).

[S45] Wang, Y. *et al.* Room temperature magnetization switching in topological insulator-

ferromagnet heterostructures by spin-orbit torques. *Nat. Commun.* **8**, 1364 (2017).

[S46] Hikami, S., Larkin, A. I. & Nagaoka, Y. Spin-orbit interaction and magnetoresistance in the two dimensional random system. *Prog. Theor. Phys.* **63**, 707–710 (1980).

[S47] Bergmann, G. Physical interpretation of weak localization: A time-of-flight experiment with conduction electrons. *Phys. Rev. B* **28**, 2914 (1983).

[S48] Bardarson, J. H. & Moore, J. E. Quantum interference and Aharonov–Bohm oscillations in topological insulators. *Rep. Prog. Phys.* **76**, 056501 (2013).

[S49] Zhang, J., Velev, J. P., Dang, X. & Tsymbal, E. Y. Band structure and spin texture of Bi_2Se_3 $3d$ ferromagnetic metal interface. *Phys. Rev. B* **94**, 014435 (2016).

[S50] Hsu, Y.-T., Park, K. & Kim, E.-A. Hybridization-induced interface states in a topological-insulator–ferromagnetic-metal heterostructure. *Phys. Rev. B* **96**, 235433 (2017).

[S51] Li, P., Appelbaum, I. *et al.* Interpreting current-induced spin polarization in topological insulator surface states. *Phys. Rev. B* **93**, 220404 (2016).

[S52] Zhu, L., Ralph, D. C. & Buhrman, R. A. Highly efficient spin-current generation by the spin Hall effect in $Au_{1\text{-}x}Pt_x$. *Phys. Rev. Applied* **10**, 031001 (2018).

[S53] Wu, H. *et al.* Room-temperature spin-orbit torque from topological surface states. *Phys. Rev. Lett.* **123**, 207205 (2019).

[S54] Chi, Z. *et al.* The spin Hall effect of Bi-Sb alloys driven by thermally excited Dirac-like electrons. *Sci. Adv.* **6**, eaay2324 (2020).

Supplementary Table I. SOTs in magnetic heterostructures. The characterization method, the resistivity of the SOT source materials ρ, the dimensionless DL-torque efficiency ξ_{DL}, the DL-torque efficiency per electric field ξ^{E}_{DL}, and the claimed origin accounted for the SOT generation are summarized. All the parameters were measured at room temperature. Here, the dimensionless DL-torque efficiency ξ_{DL} corresponds to the effective spin Hall angle. SHH, ST-FMR, HLS, and PH stands for the second harmonic Hall, the spin-torque ferromagnetic resonance, the magnetic hysteresis loop shift, and the DC planar Hall techniques, respectively. TSSs is topological surface states.

Structure	Method	ρ ($\mu\Omega$ cm)	ξ_{DL}	ξ^{E}_{DL} (10^5 Ω^{-1} m^{-1})	claimed origin
Co/Pt [16]	SHH	50	0.12	2.4	bulk SOT
Ta/$Co_{40}Fe_{40}B_{20}$ [8]	ST-FMR	190	-0.15	-0.79	bulk SOT
Co/$Au_{0.25}Pt_{0.75}$ [52]	ST-FMR	80	0.35	4.4	bulk SOT
$Co_{40}Fe_{40}B_{20}$/Bi_2Se_3 [45]	ST-FMR	4115	1.75	0.43	TSSs-origin
$Co_{77}Tb_{23}$/(Bi, Sb)$_2$Te$_3$ [43]	HLS	4020	0.4	0.1	TSSs-origin
$Co_{72}Tb_{28}$/$Bi_{0.1}Sb_{0.9}$ (This work)	SHH	389	0.51	1.3	bulk SOT
$Ni_{81}Fe_{19}$/$Bi_{0.1}Sb_{0.9}$ (This work)	ST-FMR	365	0.1	0.28	bulk SOT
CoFeB/Ti/SnTe [53]	SHH	1835	-1.41	-0.77	TSSs-orign
$Co_{20}Fe_{60}B_{20}$/Bi/(Sb/Bi)$_n$ [54]	SHH	1000	1.2	1.2	bulk SOT
CoFeB/Bi_xSe_{1-x} [44]	PH	12821	18.62	1.5	TSSs-orign
